# Chemical Characterization of the Essential Oil Compositions of *Mentha spicata* and *M. longifolia* ssp. *cyprica* from the Mediterranean Basin and Multivariate Statistical Analyses

**DOI:** 10.3390/molecules29091970

**Published:** 2024-04-25

**Authors:** Hasan İsfendiyaroğlu, Azmi Hanoğlu, Duygu Yiğit Hanoğlu, Fehmi B. Alkaş, Kemal Hüsnü Can Başer, Dudu Özkum Yavuz

**Affiliations:** 1 Department of Phytotherapy, Faculty of Pharmacy, Near East University, Nicosia 99138, Cyprus; 2 Department of Pharmacognosy, Faculty of Pharmacy, Near East University, Nicosia 99138, Cyprus; azmi.hanoglu@neu.edu.tr (A.H.); kemalhusnucan.baser@neu.edu.tr (K.H.C.B.); 3 Department of Pharmaceutical Botany, Faculty of Pharmacy, Near East University, Nicosia 99138, Cyprus; duygu.yigithanoglu@neu.edu.tr (D.Y.H.); dudu.ozkum@neu.edu.tr (D.Ö.Y.); 4 Department of Toxicology, Faculty of Pharmacy, Near East University, Nicosia 99138, Cyprus; fehmibalkas@gmail.com

**Keywords:** *Mentha longifolia* ssp. *cyprica*, *Mentha spicata*, essential oil, endemic, Cyprus, principal component analysis (PCA), hierarchical cluster analysis (HCA)

## Abstract

This present study aims to characterize the essential oil compositions of the aerial parts of *M. spicata* L. and endemic *M. longifolia* ssp. *cyprica* (Heinr. Braun) Harley by using GC-FID and GC/MS analyses simultaneously. In addition, it aims to perform multivariate statistical analysis by comparing with the existing literature, emphasizing the literature published within the last two decades, conducted on both species growing within the Mediterranean Basin. The major essential oil components of *M. spicata* were determined as carvone (67.8%) and limonene (10.6%), while the major compounds of *M. longifolia* ssp. *cyprica* essential oil were pulegone (64.8%) and 1,8-cineole (10.0%). As a result of statistical analysis, three clades were determined for *M. spicata*: a carvone-rich chemotype, a carvone/*trans*-carveol chemotype, and a pulegone/menthone chemotype, with the present study result belonging to the carvone-rich chemotype. Carvone was a primary determinant of chemotype, along with menthone, pulegone, and *trans*-carveol. In *M. longifolia*, the primary determinants of chemotype were identified as pulegone and menthone, with three chemotype clades being pulegone-rich, combined menthone/pulegone, and combined menthone/pulegone with caryophyllene enrichment. The primary determinants of chemotype were menthone, pulegone, and caryophyllene. The present study result belongs to pulegone-rich chemotype.

## 1. Introduction

Lamiaceae, the sixth largest family among the angiosperms consisting of 236 genera with over 7000 species, is composed of conventionally used medicinal plants [1]. Lamioideae and Nepetoideae are two of the most prevalent subfamilies among the total of 11 subfamilies of the Lamiaceae family [2]. The genus *Mentha* L., belonging to the Nepetoideae subfamily, consists of 24 accepted species worldwide [3,4]. *Mentha* spp., well known as “mint”, is reported to have anti-inflammatory, sedative, antioxidant, antibacterial, and antifungal effects along with several traditional uses [5]. One of the popular plants in this genus is *M. spicata* L., which is used worldwide for medicinal and culinary purposes [6]. In Cyprus, the family Lamiaceae is represented by 32 genera, and the genus *Mentha* is represented by four species; *M. aquatica* L., *M. pulegium* L., *M. spicata* L., and *M. longifolia* (L.) Huds [7,8].

The essential oil components of *M. spicata* have been extensively reviewed [6,9,10]. The essential oil composition studies have shown a variety of the major compounds in oils of *M. spicata* collected from the Mediterranean region [11,12,13,14,15,16,17,18,19,20]. Previously, *M. spicata* ssp. *spicata* oils from Turkey have been reported as rich in menthone/isomenthone, *trans*-sabinene hydrate/carvone/terpinen-4-ol, and 1,8-cineole/linalool/carvone, respectively. It was also summarized in the same article that the chemotypes of *M. spicata* growing in the Mediterranean basin until that day were piperitone oxide-rich, piperitenone oxide-rich, carvone and/or dihydrocarvone-rich, dihydrocarveol-rich, linalool-rich, and pulegone/menthone/isomenthone-rich oils [11]. There is only one report on the essential oil composition of *M. spicata* from Cyprus. The main compounds reported are carvone (71.3%) and limonene (12.5%) [12]. However, there exists a general lack of comprehensive chemotaxonomic studies using the existing literature, with previous reviews reporting the essential oil compositions of their sampling volume, but no classifications were made between the samples apart from the original classifications made by the source literature utilized in each review study [6,9,10].

The essential oil compositions of *M. longifolia* were also previously reported, revealing that in the reported literature, there exists discrepancies and variations in the reported major components of essential oils. Among those reported as major compounds of *M. longifolia* essential oil are mainly pulegone, 1,8-cineole, menthone, menthol, carvone, limonene, piperitone, piperitenone oxide [16,21,22,23,24,25,26,27]. Chemotaxonomic research on this species is not forthcoming, with there existing only three studies with relatively large sample numbers in the previous literature, with none subjecting the samples to extensive analyses to solidify chemotaxonomic classification onto a statistical foundation [11,28,29]. This is exacerbated by the fact that *M. longifolia* appears to exhibit heterogeneous essential oil composition, with significant numbers of chemotypes being identified in each previous study. However, these chemotype identifications rely on personal observational deductions from the raw data of the researchers, and, as such, they are highly subjective. An objective, statistical method is more reliable for high-fidelity, high-accuracy determination of chemotypes than any subjective measure. A recent review has concatenated and summarized these chemotypes, stressing the lack of phytochemical studies on the essential oil composition of *M. longifolia* [30]. There is only one report on the essential oil composition of *M. longifolia* ssp. *cyprica* with pulegone (71.5%), 1,8-cineole (9.5%), menthone (5.0%), and limonene (3.4%) as major components [26].

There exists a large number of subspecies and variations in the essential oil composition among *Mentha* species in the Mediterranean Basin. Therefore, it is reasonable to think that there exist distinct chemotypes that can be identified with large-scale data processing, by employing statistical methods. This present study aims to characterize the essential oil composition of the aerial parts of *M. spicata* and *M. longifolia* ssp. *cyprica*, and to perform multivariate statistical analysis, principal component (PCA) and hierarchical cluster analyses (HCA), by comparing with the existing literature, emphasizing the literature published within the last two decades, conducted on both species growing within the Mediterranean Basin. Due to the endemic nature of *M. longifolia* ssp. *cyprica*, it was compared with *M. longifolia*. This study, therefore, uses statistical methods, for the first time as far as the authors know, to identify and establish chemotypes at a higher precision in the Mediterranean Basin.

## 2. Results and Discussion

The essential oils of *M. spicata* and *M. longifolia* ssp. *cyprica* were isolated by hydrodistillation and analyzed for chemical characterization using simultaneous GC-FID and GC/MS. The yields of the essential oils were calculated on a dry weight basis as 4.0% and 3.0%, respectively. Overall, 32 and 22 identified compounds were detected, comprising the total of essential oils. The major compounds of *M. spicata* were determined as carvone (67.8%) and limonene (10.6%), while *M. longifolia* ssp. *cyprica* contained pulegone (64.8%), and 1,8-cineole (10.0%), respectively. Table 1 shows the detailed essential oil compositions of the aerial parts of *M. spicata* and the endemic *M. longifolia* ssp. *cyprica* from Northern Cyprus.

**Figure 1:**
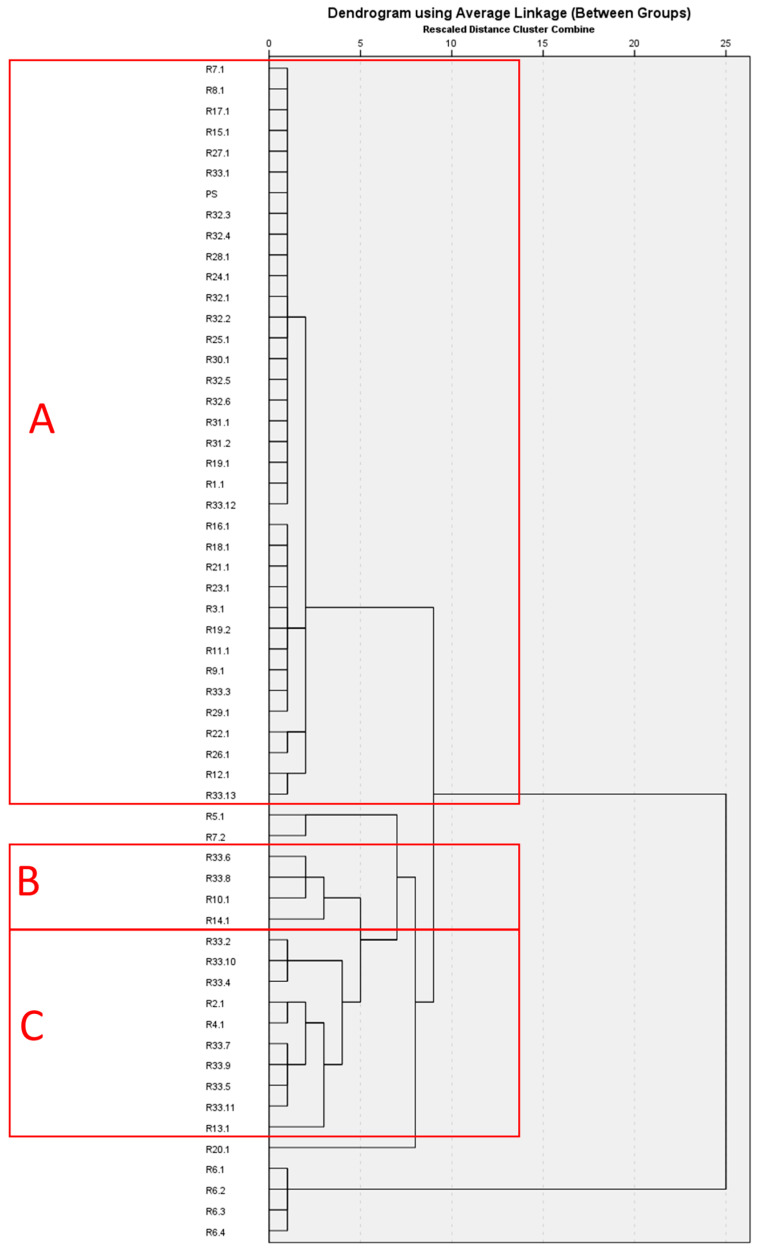
Hierarchical cluster analysis (HCA) of essential oil compositions of *M. spicata* from the Mediterranean Basin. A, B, and C refer to the clades identified by HCA (PS: present study, RX.Y refers to X: reference used, Y: sample within reference), references: R1 [15], R2 [35], R3 [36], R4 [20], R5 [16], R6 [17], R7 [34], R8 [37], R9 [38], R10 [13], R11 [39], R12 [40], R13 [41], R14 [42], R15 [43], R16 [44], R17 [45], R18 [46], R19 [47], R20 [20], R21 [48], R22 [49], R23 [50], R24 [51], R25 [52], R26 [53], R27 [54], R28 [55], R29 [56], R30 [12], R31 [57], R32 [19], R33 [14].

The HCA results of the essential oils analyses from the present study and related studies of *Mentha spicata* from within the Mediterranean region are given in Figure 1. The results indicate the presence of three major clades (given as red squares A–C). A linalool-rich chemotype (ranging between 86.7% and 93.9%) from Greece was composed of the same population sampled at different times [17]. Another outlier is also from Greece, with an unusual piperitone epoxide and piperitenone oxide (23.0% and 41.0% of total essential oil, respectively) dominant chemotype [34]. The single sample comprising another outlier was a *cis*-carvone oxide (44.1%) chemotype, with dihydrocarvone also present at 8.9% [15].

The three major clades are A–C, with A and C being closer in chemical makeup to each other than B. A and C are both carvone-bearing chemotypes, with the former containing it at an average of 60.3% and the latter at 20.8%. Clade A, which was populated with the highest number of samples, was composed of a total of 36 samples [12,13,14,15,19,34,36,37,38,39,40,43,44,45,46,47,48,49,50,51,52,53,54,55,56,57]. Clade B was composed of a total of 4 samples [13,14,42] and clade C was composed of a total of 10 samples [14,20,35,41]. Clades A and C differ from each other in not only carvone, but other constituents as well, primarily with *trans*-carveol, which is present in significantly higher concentrations in C at approximately 37.0% than it is in A, at 11.2%. Therefore, the two clades are differentiated from each other by reduced carvone and increased *trans*-carveol in C compared to A. Clade B is a combined menthone/pulegone/menthol chemotype, with average composition of 23.6% menthone and 19.6% pulegone, along with 7.16% menthol.

The *M. spicata* essential oil sample obtained from the present study was placed in Clade A, indicating that it belongs to the carvone-rich chemotype dominated by this compound. The other study from Cyprus that also characterized *M. spicata* essential oil [17] was placed into the same clade, with low Euclidean distancing, indicating that they belong to the same chemotype and are of similar composition to the present study.

The three major clades (A, B, and C) were also subjected to ANOVA to determine statistically significant differences in essential oil content. Isolated samples were not considered for ANOVA due to limited sample sizes. The distinguishing feature of the three clades was found to be the carvone content, with clade A having the greatest concentration, followed by C, and the least carvone being present in B (*p* < 0.001). Therefore, carvone concentration in essential oil can be considered to be a major determinant in the distinction of these clades from each other. It was determined that 1,8-cineole and limonene contents were not significantly different among the three groups (*p* > 0.05) and, therefore, are not significant determinants of chemotype among these three clades.

Pulegone, *cis*-isopulegone, menthol, and menthone content of the essential oil were both determined to be a statistically significant determinant of chemotype, with A and C being similar to each other (*p* > 0.05), but B being significantly different from either (*p* < 0.001). All of these phytochemicals were present in higher concentrations in essential oils obtained from samples in B, compared to those in A or C.

A statistically significant increase was observed in the concentration of the essential oil of 2-hydroxy-3-(3-methyl-2-butenyl)-3-cyclopenten-1-one in C compared to A (*p* < 0.001) at an average concentration of 7.2% in C compared to an average of 0.1% in A, providing another differentiating characteristic between the two clades [14]. However, the identification of this compound was determined to be irrelevant since it was not reported from any natural source in *M. spicata* in the previous literature.

In light of these statistical analyses, as explained above, it can be suggested that three definitive and four isolated samples can be associated with *M. spicata*. The isolated samples are putative chemotypes due to the presence of a single sample in each category; therefore, no definitive assertions can be made concerning their chemotypic uniqueness. However, much more definitive deductions can be made about clades A–C, which, according to the PCA, HCA, and ANOVA discussed herein, can be divided into three chemotypes: a carvone-rich chemotype with a simple majority of carvone associated with clade A, a carvone-poor chemotype that also features enrichment in menthol, menthone, pulegone, and *cis*-isopulegone, associated with clade B, and a carvone-rich chemotype that is not as rich in carvone as clade A but containing higher concentrations of *trans*-carveol (Table 2).

**Figure 2:**
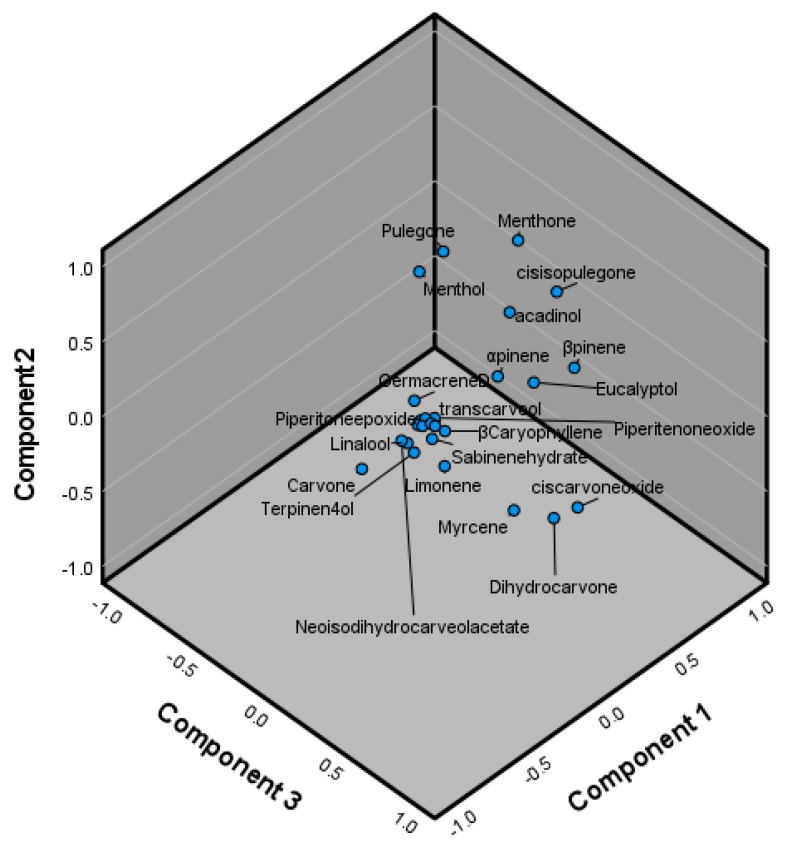
Principal component 3D graph for Component 1 (PC1), Component 2 (PC2), and Component 3 (PC3) in *Mentha spicata.* Eucalyptol = 1,8-cineole, Corylone = 2-Hydroxy-3-(3-methyl-2-butenyl)-3-cyclopenten-1-one.

The results indicate that among the *M. spicata* essential oil samples obtained from references, as well as the current study, there exists a significant variation in the essential oil composition, with certain possible clusterings (Figure 2). Among these, linalool, carvone, pulegone, and menthone appear to be significant components contributing to essential oil variation, with some others, such as limonene and 1,8-cineole, contributing to a lesser degree (Table 2).

**Figure 3:**
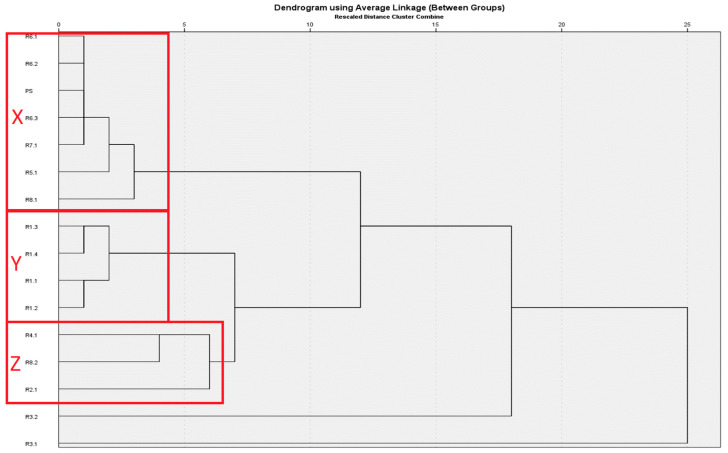
Hierarchical cluster analysis (HCA) of essential oil compositions of *Mentha longifolia* from the Mediterranean Basin. X, Y, and Z refer to the clades identified by HCA. (PS: present study, Ra.b refers to a: reference used, b: sample within reference), references: R1 [23], R2 [24], R3 [16], R4 [26], R5 [25], R6 [22], R7 [21], R8 [27].

The samples within *M. longifolia* were divided into three definitive clades and two putative clades, made of single members. The putative clades were disregarded for further statistical analyses. The definitive clades were named X, Y, and Z (Figure 3). Clade X was composed of a total of seven samples [21,22,25,27]. Clade Y was composed of a total of four samples [28]. Clade Z was composed of a total of three samples [24,26,27].

Clade X corresponds to a pulegone-rich clade with strong enrichment in pulegone (56.9% averaged), and low menthone content at approximately 6.3%. The current study sample is in clade X, with 64.8% pulegone and 7.6% menthone content. Clade Y corresponds to a menthone/pulegone chemotype that displays reduced pulegone (14.4% on average) but also displays enrichment in menthone (25.2% on average). Clade Z is characterized by a combined menthone/pulegone chemotype (12.6% and 18.3%, respectively) and slight caryophyllene enrichment (2.8% on average) (Table 3).

**Figure 4:**
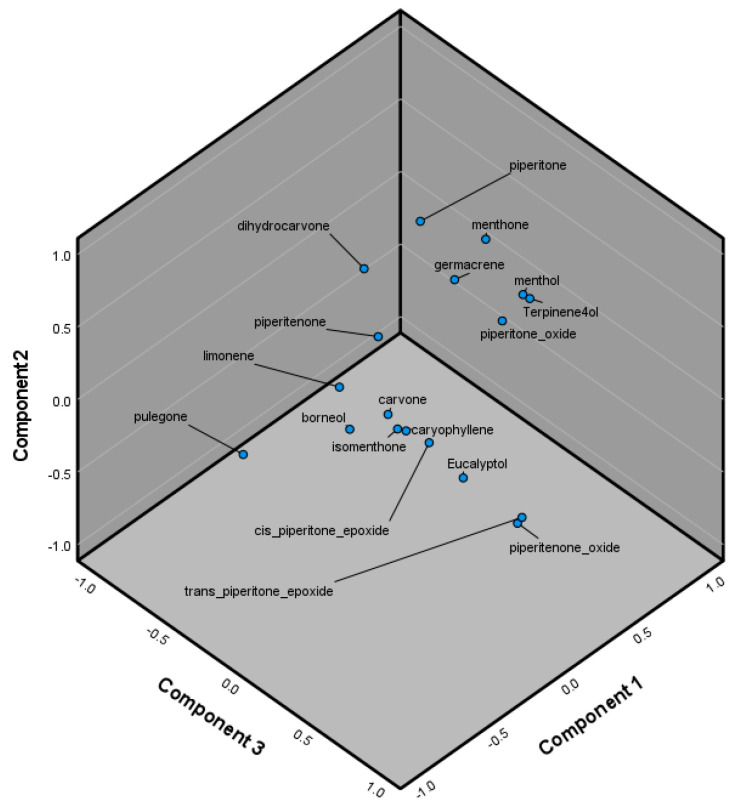
Principal component 3D graph for Component 1 (PC1), Component 2 (PC2), and Component 3 (PC3) in *Mentha longifolia*.

ANOVA revealed that menthone was a major determinant of chemotype, with clades X and Z on one hand and Y on the other, being significantly different in menthone concentrations of their essential oils (*p* < 0.05). X and Z did not have a significant difference in menthone concentration (*p* > 0.05). On the other hand, clade X was differentiated from Y and Z with a significant difference in pulegone concentration (*p* < 0.05), whereas Y and Z did not have a significant difference in pulegone content. Finally, there existed a statistically significant difference in the caryophyllene concentration in clade Z compared to clades X and Y (*p* < 0.05). Clades X and Y did not have significantly different caryophyllene content (*p* > 0.05). These findings were corroborated by the PCA conducted on *M. longifolia*, which indicated menthone, pulegone, and caryophyllene as significant contributors to variation among samples. Terpinen-4-ol and piperitone oxide could not be determined, due to inability to establish homogeneity of variances as a prerequisite for ANOVA (Table 3, Figure 4).

The present study results demonstrate the utility of multivariate statistical analysis of essential oil for the determination of chemotypic taxonomy. These novel methods, with their higher discretionary power, allow for more reliable classification of samples into existing chemotypes. Such applications are important when, for example, cultivating plants for medicinal or industrial purposes, so that the essential oil composition, and therefore the utility of plants from a medicinal or industrial standpoint, can be more definitively ascertained, increasing safety for the former, and yield, and efficiency for the latter. As an example, a recent study, also utilizing the principles of HCA and PCA to compare their samples, discovered previously undescribed chemotypes of *Mentha* sp. [58].

The statistical determination of chemotype Is also highly important in chemotaxonomy from a purely scientific perspective. In the present study, the present sample was chemotaxonomically classified purely on the basis of the HCA classification. While such a result could also be obtained from a subjective determination of the chemotype, there may always be confounding factors that a scientist may ignore or miss. For example, the *M. longifolia* analysis in this study indicated that the slight enrichment in caryophyllene is a determining factor in clade Z, despite the caryophyllene concentration not exceeding 3%. It, thus, demonstrates that different compounds can be strong determinants of chemotype even in small concentration differences, which may not necessarily be all that obvious to a subjective determination by a human. This chemotaxonomy determination based on statistical differences, regardless of the concentration scale in question, opens a new dimensionality to chemotype determination, and allows for the distinction between chemotypes that are superficially similar in the composition of the major substances, but may exhibit subtle differences in their composition, therefore comprising two different, albeit closely related, chemotypes.

These differences in composition are especially important in the medicinal and industrial cultivation of such plants, where the toxicological profile, which depends on not only the concentration, but also the specific toxicity of the compounds in question, chemotypic differences between a chemotype that does not contain any toxic compounds above threshold limits, and a chemotype that has a slightly higher concentration of a highly toxic compound, becomes crucial from a safety perspective, thus requiring the distinction of chemotypes based on chemical composition.

## 3. Materials and Methods

### 3.1. Plant Material

The collected plant materials were identified by K.H.C. Başer and D. Özkum Yavuz, according to the Flora of Cyprus [13]. Aerial parts of the cultivated *M. spicata* (Flowering period: July–November)*,* and natural *M. longifolia* ssp. *cyprica* (flowering period: June–November) were collected from Cengizköy-Lefka/Northern Cyprus on 1 July 2022 during the flowering stage. They were separately air-dried in the shade. Voucher specimens are kept at the Herbarium of the Near East University, Turkish Republic of Northern Cyprus (NEUN) with the voucher numbers NEUN 20001 and 20002.

### 3.2. Isolation of Essential Oils

One hundred grams of air-dried samples were separately distilled for 3 h using a Clevenger-type apparatus by hydrodistillation. The resulting essential oil was stored at 4 °C until further analyses. The oil yields were calculated as *v*/*w* on a dry weight basis.

### 3.3. Selection of Mentha spicata and M. longifolia Essential Oils for Multivariate Analyses

The samples for comparison were selected from previous research conducted on *Mentha spicata* and *M. longifolia* within the Mediterranean Basin. All samples were restricted to the Mediterranean Basin to prevent inevitable divergence in plant physiology due to divergent edaphic and environmental factors. Previous research was also limited to approximately the last 20 years so that differences in available technological tools and research methodologies could be minimized. Studies that indicated raw percentile values were taken into account, with those that gave mean ± SD also excluded from analysis.

### 3.4. GC-FID Analysis

The GC/MS analysis was carried out with an Agilent 5977B GC-MSD system (Santa Clara, CA, USA). Innowax FSC column (Agilent, 60 m × 0.25 mm, 0.25 mm film thickness) was used with helium as carrier gas (0.8 mL/min). GC oven temperature was kept at 60 °C for 10 min and programmed to 220 °C at a rate of 4 °C/min, and kept constant at 220 °C for 10 min and then programmed to 240 °C at a rate of 1 °C/min. Split ratio was adjusted at 40:1. The injector temperature was set at 250 °C. Mass spectra were recorded at 70 eV. Mass range was from *m*/*z* 35 to 450. FID results were used to report the characterized compounds’ relative percentages (%) [59].

### 3.5. GC/MS Analysis

The analysis was carried out using an Agilent 7890B GC system. The integrated FID detector temperature was 300 °C. To obtain the same elution order with GC/MS, simultaneous auto-injection was performed on a duplicate of the same column applying the same operational conditions. Relative percentage amounts of the separated compounds were calculated from FID chromatograms. Identification of the essential oil components was carried out by comparison of their relative retention times (RRT) with those of authentic samples or by comparison of their linear retention index (LRI) to a series of *n*-alkanes. Computer matching against commercial (Wiley GC/MS Library, NIST Library) and in-house “Başer Library of Essential Oil Constituents” built up by genuine compounds and components of known oils, as well as MS literature data, was used for the identification [59].

### 3.6. Statistical Analysis

All relevant data were imported to IBM SPSS Statistics v27.0 (International Business Machines (IBM) Corporation, Armonk, NY, USA). Principal component analysis (PCA) was performed using the correlation matrix method with sequential eigenvalues selected based on the introduction of “kinks” in the scree plot. The varimax rotation method was employed to improve the correlation between chemical constituents and principal components. Correlation matrices were employed to ascertain the effect of different constituents on chemotypes. Hierarchical cluster analysis (HCA) was performed using the squared Euclidean distance between-groups linkage method using agglomeration schedules. The dendrograms were produced from the HCA using this data. Only components that were deemed major by the authors of at least one of the references cited herein were included in the PCA and HCA. Previous studies from the Mediterranean Basin were included in the present study as references, with those outside of the Mediterranean Basin excluded from the study. The clades as determined by HCA were subjected to one-way analysis of variance (ANOVA) to confirm chemotypic differences, with Levene’s test employed to test for homogeneity of variances, and Bonferroni’s post hoc test was utilized to ascertain differences between identified clades.

## 4. Conclusions

The essential oil composition of *M. spicata* growing in Northern Cyprus revealed that the major components were carvone (67.8%) and limonene (10.6%), while the major compounds of *M. longifolia* ssp. *cyprica* essential oil were pulegone (64.8%) and 1,8-cineole (10.0%), respectively. The multivariate analysis showed the existence of three major clades within the *M. spicata* growing in Mediterranean region, with a carvone-rich chemotype, a carvone/*trans*-carveol chemotype, and a pulegone/menthone chemotype, with the present study belonging to the carvone-rich chemotype. It was determined that carvone was a primary determinant of chemotype, along with menthone, pulegone, and *trans*-carveol. It was also determined that 1,8-cineole was not an important determinant of chemotype.

In *M. longifolia* growing in Mediterranean region, it was determined that the primary determinants of chemotype were pulegone and menthone, with three chemotypes being identified as pulegone-rich, combined menthone/pulegone, and combined menthone/pulegone with caryophyllene enrichment. The Cyprus endemic, *M. longifolia* ssp. *Cyprica,* sample analyzed in the current study is in the pulegone-rich clade.

HCA, PCA, and ANOVA all demonstrate that the *Mentha* species within the Mediterranean Basin display remarkable biodiversity. To the extent of the authors’ knowledge, this study is the first of its kind to establish the biodiversity of *Mentha* species in the Mediterranean Basin at a statistical level; therefore, the present study has pioneering value. This is especially true in consideration of the fact that *Mentha* species display remarkable diversity in essential oil composition even when the edaphic factors are highly convergent, in stable climatic conditions such as that within the Mediterranean Basin, displaying that *Mentha* is a remarkably biodiverse genus.

## Figures and Tables

**Table 1:** The essential oil compositions of the aerial parts of cultivated *M. spicata* and natural *M. longifolia* ssp. *cyprica* from Northern Cyprus.

KI	LRI	Compound Name	*M. spicata*	*M. longifolia* ssp. *cyprica*
			Relative Percentages (%)	
1008–1039 ^b^	993	α-Pinene	1.0	0.9
1012–1039 ^b^	997	α-Thujene	0.1	-
	1017	2,5-Diethyltetrahydrofuran	0.1	-
1085–1130 ^b^	1089	β-Pinene	1.2	1.7
1098–1140 ^b^	1104	Sabinene	0.6	1.0
1140–1175 ^b^	1143	Myrcene	0.7	1.1
1178–1219 ^b^	1180	Limonene	10.6	3.9
1198–1234 ^a^	1193	1,8-Cineole	4.8	10.0
1211–1251 ^b^	1212	(*Ζ*)-β-Ocimene	-	0.4
1357–1417 ^a^	1367	3-Octanol	0.3	-
1438–1474 ^a^	1444	*trans*-Sabinene hydrate	0.5	-
1440–1492 ^a^	1454	Menthone	-	7.6
1453–1525 ^a^	1481	Isomenthone	-	0.3
1495–1546 ^a^	1497	β-Bourbonene	0.8	-
1556–1600 ^a^	1567	β-Elemene	0.6	-
1583 ^a^	1568	*cis*-Isopulegone	-	0.5
1587–1597 ^a^	1577	*trans*-Isopulegone	-	0.4
1570–1617 ^a^	1579	β-Caryophyllene	0.9	1.7
1564–1630 ^b^	1585	Terpinen-4-ol	0.4	-
1602–1650 ^b^	1601	*trans*-Dihydrocarvone	1.1	-
1631–1665 ^b^	1641	Pulegone	1.1	64.8
1657–1700 ^c^	1651	Dihydrocarvyl acetate	1.0	-
1656–1690 ^a^	1660	α-Humulene	0.6	0.4
1664–1694 ^a^	1662	*trans*-Verbenol	-	0.1
1646–1741 ^a^	1676	α-Terpineol	-	0.8
1677–1731 ^a^	1677	α-Terpinyl acetate	0.2	-
1675–1723 ^a^	1684	Borneol	0.3	-
1665–1746 ^a^	1692	Germacrene D	0.5	0.9
	1703	Neodihydrocarveol	0.4	-
1699–1769 ^a^	1717	Bicyclogermacrene	0.4	0.7
1696–1748 ^a^	1725	Piperitone	-	0.4
1713–1763 ^a^	1734	Carvone	67.8	-
1710–1782 ^a^	1751	*cis*-Carvyl acetate	0.8	-
1800–1836 ^a^	1814	Calamenene	0.6	-
	1833	Carvone-1,2-oxide	0.2	-
1819–1881 ^a^	1845	*cis*-Carveol	1.7	-
1918–1956 ^a^	1928	Piperitenone	0.2	1.5
1983–1984 ^a^	1958	Piperitenone oxide	-	0.8
2034–2090 ^a^	2043	Cubenol	0.3	-
2090–2153 ^a^	2110	Spathulenol	0.1	0.2
2175–2259 ^a^	2216	α-Cadinol	0.2	-
		Monoterpene hydrocarbons	14.2	9.0
		Oxygenated monoterpenes	80.5	87.2
		Sesquiterpene hydrocarbons	4.4	3.7
		Oxygenated sesquiterpenes	0.6	0.2
		Others	0.4	-
		Total	100.0	100.0

KI: from literature [31] ^a^, [32] ^b^, [33] ^c^, LRI (linear retention index) calculated against *n*-alkanes series; % calculated from FID data.

**Table 2:** The rotated component (loadings) matrix of the essential oil compositions of *Mentha spicata* in the Mediterranean Basin.

Compounds	Components			
	1	2	3	4
Menthol	0.973			
Pulegone	0.960	0.101		
Menthone	0.696	0.622		
*cis*-Isopulegone	0.145	0.925		−0.151
β-Pinene		0.733		0.104
Eucalyptol (=1,8-Cineole)		0.502		
*cis*-Carvone oxide			0.916	
*trans*-Carveol			0.886	
*cis*-Carveol			0.814	
β-Caryophyllene			0.151	0.865
Germacrene D	0.110			0.773
Linalool				0.623
Dihydrocarvone	−0.219		−0.239	0.541
*p*-Cymene				
Myrcene	−0.136			
α-Pinene		0.420		−0.159
Corylone (=2-Hydroxy-3-(3-methyl-2-butenyl)-3-cyclopenten-1-one)	−0.117			
α-Cadinol	0.156	0.606		−0.121
Carvone	−0.470	−0.369		−0.237
Sabinene hydrate	−0.140		−0.110	
Dihydrocarveol		−0.104	0.167	−0.120
Piperitenone oxide				−0.130
Terpinen-4-ol	−0.137	−0.143		
Limonene	−0.338		−0.259	
Neoiso-Dihydrocarveol acetate	−0.156	−0.111	−0.132	−0.127

**Table 3:** The rotated component (loadings) matrix of the essential oil compositions of *Mentha longifolia* in the Mediterranean Basin.

	Components					
	1	2	3	4	5	6
Terpinen-4-ol	0.954					
Menthol	0.944		−0.100		−0.106	
Menthone	0.821	0.359	−0.234	−0.214	−0.151	
Piperitone oxide	0.735					
Pulegone	−0.549	−0.368	−0.533	−0.447		−0.134
Dihydrocarvone	−0.168	0.962				0.102
Piperitone	0.269	0.945	−0.132			
Germacrene D	0.169	0.851	0.204		−0.311	−0.135
Piperitenone	−0.117	0.490		0.460	0.478	−0.244
*trans*-Piperitone epoxide	−0.131		0.968		−0.125	
Piperitenone oxide	−0.157		0.963	−0.101		0.129
Caryophyllene	−0.464	0.468	0.503	0.250	0.420	0.127
Carvone				0.978		
Limonene	−0.253	0.146	−0.167	0.898	−0.214	
Isomenthone		−0.130		−0.113	0.945	0.183
Borneol	−0.197	−0.174	−0.152	−0.151	0.774	
*cis*-Piperitone epoxide			0.263		0.144	0.893
Eucalyptol (=1,8-cineole)		−0.106	0.523	−0.309		−0.713

## Data Availability

The data used to support the findings of this study are included within the article. For further data, they are available from the corresponding author upon request.
